# The Use of CRISPR/Cas9 as a Tool to Study Human Infectious Viruses

**DOI:** 10.3389/fcimb.2021.590989

**Published:** 2021-08-27

**Authors:** Huafeng Lin, Gang Li, Xiangwen Peng, Aimin Deng, Lei Ye, Lei Shi, Tuanmei Wang, Jun He

**Affiliations:** ^1^Changsha Hospital for Maternal and Child Health Care of Hunan Normal University, Changsha, China; ^2^Institute of Food Safety and Nutrition, Jinan University, Guangzhou, China; ^3^Institute of Biomedicine and Department of Cell Biology, Jinan University, Guangzhou, China

**Keywords:** CRISPR/Cas9, delivery mode, HIV, HBV, HPV, infectious viruses, off-target effects

## Abstract

Clustered regularly interspaced short palindromic repeats (CRISPR) systems are a set of versatile gene-editing toolkit that perform diverse revolutionary functions in various fields of application such as agricultural practices, food industry, biotechnology, biomedicine, and clinical research. Specially, as a novel antiviral method of choice, CRISPR/Cas9 system has been extensively and effectively exploited to fight against human infectious viruses. Infectious diseases including human immunodeficiency virus (HIV), hepatitis B virus (HBV), human papillomavirus (HPV), and other viruses are still global threats with persistent potential to probably cause pandemics. To facilitate virus removals, the CRISPR/Cas9 system has already been customized to confer new antiviral capabilities into host animals either by modifying host genome or by directly targeting viral inherent factors in the form of DNA. Although several limitations and difficulties still need to be conquered, this technology holds great promises in the treatment of human viral infectious diseases. In this review, we will first present a brief biological feature of CRISPR/Cas9 systems, which includes a description of CRISPR/Cas9 structure and composition; thereafter, we will focus on the investigations and applications that employ CRISPR/Cas9 system to combat several human infectious viruses and discuss challenges and future perspectives of using this new platform in the preclinical and clinical settings as an antiviral strategy.

## Introduction

Viruses are the deep cause for numerous acute and chronic diseases, some of which lead to severe situations, like the recent coronavirus disease 2019 (COVID-19) pandemic. However, some of them just produce minor diseases, like herpes simplex viruses. Currently, serious viral infectious illnesses such as human immunodeficiency virus (HIV), hepatitis B virus (HBV), and human papillomavirus (HPV) are potentially threating the human health and global stability (Morens and Fauci, 2013). They undoubtedly increase the socioeconomic burden on the public health systems throughout the world (Doerflinger et al., 2017). Compared to other health-relevant infectious viruses including herpes simplex virus, the three abovementioned viruses are more dangerous to human; once infected, it is difficult to cure, as the success rates with medical therapy are relatively lower. The therapy for fighting against viral infections is a challenging project, due to the overconsumption of cellular resources by viruses and the formation of latent viral reservoirs in the hosts (White et al., 2015). Moreover, many human viruses are capable of generating mutant strains to escape and even jump between different species resulting in pandemics (Parrish et al., 2008). Therefore, a series of antiviral strategies, such as synthesis drugs (Villa et al., 2017), herbal medicines (Castilla et al., 2010), animal-based medicines (Costa-Neto, 2005), antibody-based drugs (Kuprash et al., 2017), and genetically engineered drugs (Zündorf and Dingermann, 2000), have entered the preclinical and clinical fields one after another. Among the strategies, clustered regularly interspaced short palindromic repeats (CRISPR)/Cas genome editing technique, as a landmark discovery, has entered the field of biomedical research and gene therapy research, which holds great promise for tackling serious human infectious viruses.

Since 2013, the success of genome modifications *via* CRISPR/Cas9 apparatus in cultured human cells (Cho et al., 2013; Jinek et al., 2013; Mali et al., 2013) has opened up a new route for gene therapy in biomedical research. Gene editing is a combinational process of introducing site-specific DNA cleavages by nucleases and wielding the natural cellular pathways to repair the DNA breaks. Exogenous DNA double-strand breaks can be created in the genomes by means of various genome engineering platforms such as meganuclease (Maeder and Gersbach, 2016), zinc-finger nucleases (ZFNs), transcription activator-like effector nucleases (TALENs), and CRISPR/Cas nuclease systems (Ran et al., 2013; Hryhorowicz et al., 2017). Then, cell DNA repairs initiated by DNA lesions are completed through the homology-directed repair (HDR) pathway with repair templates or the nonhomologous end joining (NHEJ) pathway without repair templates (Mao et al., 2008). To date, CRISPR/Cas genome editing system has been developed as a robust instrument for targeted gene modifications in a broad range of animal species, gut microbiota (Citorik et al., 2014; Cresci et al., 2020), and their invading viruses (Ashfaq and Khalid, 2020), which cause changes in relationships between host and viruses. With the expansion of science interests in gene editing research, a new class of medicine based on CRISPR/Cas9 editing technology is entering clinical era for the treatment of viral infections (Hirakawa et al., 2020).

In this review, we will first outline a basic biological feature of CRISPR/Cas9 that focused on its structure and composition; then, we will prominently present the application investigations that employed CRISPR/Cas9 system to combat several human infectious viruses. Lastly, we will also discuss the known and potential limitations of CRISPR/Cas9 gene editing platform including off-target effects, delivery challenges, Cas9 cleavage activity, resistance to CRISPR/Cas9, viral escape problems, and ethical concerns.

## Basic Biological Features of CRISPR/Cas9 Machinery

**Figure 1:**
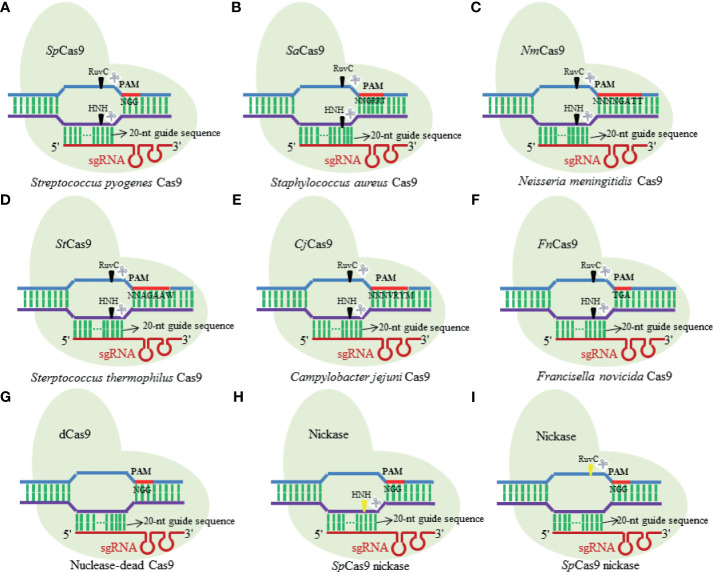
Diagram illustrating several structural variants and orthologues of natural Cas9 nuclease. **(A)** *Streptococcus pyogenes* Cas9; **(B)** *Staphylococcus aureus* Cas9; **(C)** *Neisseria meningitidis* Cas9; **(D)** *Sterptococcus thermophilus* Cas9; **(E)** *Campylobacter jejuni* Cas9; **(F)** *Francisella novicida* Cas9; **(G)** nuclease-dead Cas9; **(H)** SpCas9 nickase; **(I)** SpCas9 nickase.

First described by Ishino et al. in 1987, CRISPR is a range of DNA repeat sequences with uncertain origin and unknown function in the *Esherichia coli* genome (Ishino et al., 1987). The CRISPR/Cas complexes existing in the prokaryotic organisms confer resistance to new incoming genetic elements such as plasmids, phages, and viruses (Jinek et al., 2012). According to the Cas protein contents and its amino acid sequences, the CRISPR/Cas systems have been initially artificially divided into three major types, namely, type I, type II, and type III (Wiedenheft et al., 2012; Chylinski et al., 2014). Until recently, three additional types of CRISPR/Cas system (types IV–VI) have been identified across bacterial genomes (Bayat et al., 2018), of which both type II and type V CRISPR/Cas systems are the similar apparatuses that consist of only single subunit RNA effector (Cas9 and Cas12, respectively) (Khadempar et al., 2019). The type II CRISPR/Cas system that is commonly known as CRISPR/Cas9 is a binary complex of endonuclease Cas9 and two small guide RNAs (gRNAs) [CRISPR RNA (crRNA) and transactivating CRISPR RNA (tracrRNA) (Gebre et al., 2018)] (Saayman et al., 2015), extensively used for RNA-programmable genome manipulating purpose in most cases. It just needs the optimization of Cas9 expression (Wright et al., 2015) and the matched design of gRNA to successfully function in its editing roles in cell genomes (Jiang et al., 2015). In such type of nuclease systems, the gRNA recognized by Cas9 proteins in a sequence independent manner (Nishimasu et al., 2014) directs Cas9 to recognize and cleave target specific DNA sequences with a short protospacer adjacent motif (PAM) of 17–20 nucleotides *via* the Watson–Crick base-pairing interactions (Garneau et al., 2010). The natural CRISPR/Cas9 systems possess a variety of structural variants and orthologues (**Figure 1**) (Ran et al., 2013; Lin et al., 2019), of which *Streptococcus pyogenes* Cas9 (SpCas9) and *Staphylococcus aureus* (SaCas9) are the two types that are widely used for research purposes. Furthermore, Cas9 nucleases from different bacterial species recognize different PAM sequences for seeking targets (Lino et al., 2018), with SpCas9 using “NGG” PAM as a binding target while SaCas9 employing “NNGRRT” PAM (Xie et al., 2018).

In the last few years, the simple-designed CRISPR/Cas9 system has greatly expanded its application scopes in life science, and now, it is still in its rapidly developing stage. As an effective, highly specific and robust tool, CRISPR/Cas9 machinery holds great promise for targeting infectious viruses and removing their reservoirs to enhance human health.

## Applications of CRISPR/Cas9 to Specifically Target Infectious Viruses

**Figure 2:**
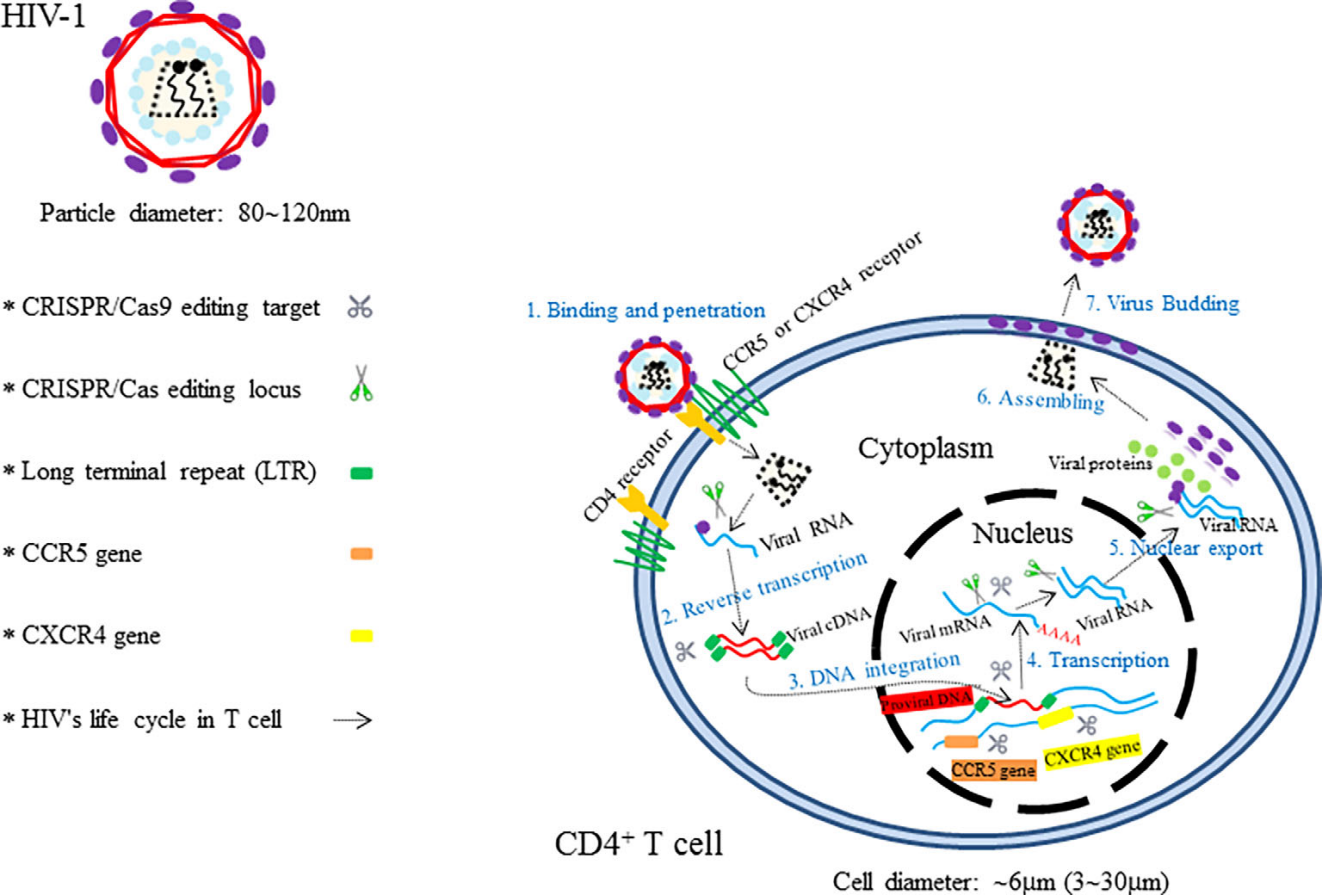
The illustration showing HIV’s invasion paths in cells and the therapeutic targets of CRISPR/Cas9.

Theoretically, the CRISPR/Cas9 technology can be used not only to target any special nucleotide sequences in human genome but also to edit the double-stranded DNA (dsDNA) of viral invaders in *in vivo* and *in vitro* system (**Figure 2**) (Soppe and Lebbink, 2017). Moreover, the technologies of Cas9 equipped with multiple single guide RNAs (sgRNAs) have enabled the Cas9 endonucleases to target several different genomic loci in a single cell (Ota et al., 2014; Zhou et al., 2014). Besides, the Cas9 variants and orthologs also give the CRISPR/Cas system many more novel functions including targeted gene mutation, transcriptional activation and inhibition, epigenetic modification, imaging of DNA loci, and single base mutation (Cong et al., 2013; Barrangou and Doudna, 2016; Mohanraju et al., 2016; Hu et al., 2018; Miller et al., 2020). Furthermore, viral eradications from cells *via* CRISPR/Cas9 machinery could be theoretically applicable to any DNA or RNA virus with a DNA intermediate in its life cycle (Doudna and Charpentier, 2014; Khalili et al., 2015; Mohammadzadeh et al., 2020). Therefore, the CRISPR/Cas9 methodology with functional diversities holds huge potential promise for targeting different developmental phases of the viral life cycle and possess the ability to mediate an effective and sustained genetic therapy against human viruses. Herein, CRISPR/Cas9-based antiviral approach to manipulate major human infectious viruses including HIV, HBV, HPV, and other viruses will be discussed.

### Human Immunodeficiency Virus

Acquired immunodeficiency syndrome (AIDS) caused by HIV infection is a kind of viral infectious disease. HIV, which mainly consists of HIV-1 and HIV-2, is an important global epidemic that requires advanced clinical remedies. According to the new report of United Nations Programme on HIV/AIDS (UNAIDS), more than 36.7 million people are infected with HIV in the whole world, and the new infection number is over 5,000 per day (Dash et al., 2019). Compared to HIV-2, HIV-1 is characterized by having higher transmissibility and pathogenicity in the human host (Campbell-Yesufu and Gandhi, 2011). Active HIV-1 reproduction *in vivo* causes severe CD4^+^ T-cell depletion, which ultimately results in the formation of the so-called chronic disease of AIDS (Alimonti et al., 2003; Doitsh et al., 2014). Great success has been achieved with the use of antiretroviral therapy (ART) and high active antiretroviral therapy (HAART) for the control of the deadly AIDS and even for lifesaving (Deng et al., 2018; Lu et al., 2018). However, these types of HIV therapeutics, designed to suppress various steps of the viral life cycle (Arribas and Eron, 2013), are still unable to cure the disease owing to the existence of permanent integration of HIV-1 into the host genome. In view of these facts, researchers have focused on the treatment of AIDS *via* CRISPR/Cas9-based gene editing systems, aiming to unlock many new possibilities for HIV-1 prevention and cure (Dampier et al., 2014). Since the first two CRISPR/Cas9-based applications in the prevention of HIV-1 have been reported by Cho and Ebina, respectively, in 2013 (Cho et al., 2013; Ebina et al., 2013), numerous studies that employ CRISPR/Cas9 technology as a method for the treatment of HIV-1/AIDS have been developed rapidly (Khalili et al., 2015). Up to now, targeting host genes and targeting viral genomes are two essential approaches for combating HIV-1 infection (Xiao et al., 2019). The attractive editing targets of CRISPR/Cas9 therapy mainly include C–C chemokine receptor 5 (CCR5) gene, C–C–C chemokine receptor 4 (CXCR4) gene, proviral DNA-encoding viral proteins, and the HIV 5′ and 3′ long terminal repeat (LTR) (**Figure 2**) (Manjunath et al., 2013; Bialek et al., 2016; Liu et al., 2017). Although Cas9/multiplexed-sgRNA technology has emerged, the use of CRISPR/Cas9 molecular scissor to precisely and jointly target two coreceptor genes, CCR5 and CXCR4, has not yet been seen in relevant reports. Here, **Table 1** lists the research studies of HIV-1 infection *via* CRISPR/Cas9 techniques for editing the aforementioned gene sites (Cho et al., 2013; Ebina et al., 2013; Hu et al., 2014; Hou et al., 2015; Wang et al., 2014; Ye et al., 2014; Liao et al., 2015; Zhu et al., 2015; Kaminski et al., 2016; Soppe and Lebbink, 2017; Liu et al., 2018; Kaushik et al., 2019; Binda et al., 2020).

**Table 1:** Applications of CRISPR/Cas9 system for gene therapy of HIV infection.

Virus Type	Target Gene	Editing System	Number of gRNA	Cell Model/Animal Model	Delivery Methods	Reference
	CCR5	Cas9/gRNA	Single gRNA	HEK293T cells	Plasmid transfection	Cho et al., 2013
HIV-1	LTR	CRISPR/Cas9	Two gRNAs	Jurkat cells, HeLa cells, T cells	Plasmid transfection	Ebina et al., 2013
HIV-1	LTR U3 region	Cas9/gRNA	Single gRNA, Multiple gRNAs	Microglial, promonocytic, and T cells	Plasmid transfection	Hu et al., 2014
HIV-1	CCR5	CRISPR/Cas9	Multiple gRNAs	293T cells, TZM.bl cells, CEMss-CCR5 cells	Lentiviral transduction	Wang et al., 2014
HIV-1	CCR5 (exon 4)	TALENs; CRISPR/Cas9	Multiple gRNAs	iPSCs	The piggyBac transposon vectors, cotransfection	Ye et al., 2014
HIV-1	CXCR4	CRISPR/Cas9	Multiple gRNAs	Ghost-CXCR4 cells, Jurkat cells and primary human CD4+ T cells	Lentivirus-mediated delivery	Hou et al., 2015
HIV-1	LTR U3, T and R region	Multiplex CRISPR/Cas9	Single gRNA, Multiple gRNAs	HEK293T cells, hPSCs	Plasmid transfection, lentiviral transduction	Liao et al., 2015
HIV-1	LTR, pol gene, and tat/rev	CRISPR/Cas9	Ten gRNAs	Jurkat cell lines	Nucleo transfection	Zhu et al., 2015
HIV	LTR U3 region	saCas9/multiplex gRNAs	Multiple gRNAs	MEFs, transgenic mice, rats	Lentiviral delivery	Kaminski et al., 2016
HIV-1	LTR	CRISPR/Cas9	Single gRNA, Multiple gRNAs	HEK293T cells, J.Lat FL cells, human T lymphoblast cells	Lentiviral transduction	Lebbink et al., 2017
HIV-1	CXCR4	CRISPR/Cas9	Multiple gRNAs	TZM-bl cells	Lipofectamine 2000	Liu et al., 2018
HIV-1	LTR	CRISPR/Cas9	Multiple gRNAs	Latent microglial cells	Magnetic delivery	Kaushik et al., 2019
HIV-1	Proviral DNA	CRISPR/Cas9	Two gRNAs	HEK 293T cells	Lentiviral transfection	Binda et al., 2020

At present, purging of the latent viral reservoirs is the biggest hurdle for the effective management of HIV infection. As observed in HIV patients who are receiving ART therapy, latent viral reservoirs that mostly attach within resting memory CD4^+^ T cells, are able to stay for as long as 60 years (Siliciano et al., 2003).

It has been about 30 years since the discovery of HIV, but there is still no effective anti-HIV vaccine available (Haynes, 2015). The “Berlin patient” has been generally recognized as the only one case cured for HIV-1 for a decade (Hutter et al., 2009; Gupta et al., 2019), and now the “London patient” would probably be the second (Gupta et al., 2020). Stem cell transplantation (SCT) is scientifically not a standard treatment method for HIV/AIDS. Viewed from these two case reports, SCT used in the two patients is originally intended for treating cancer rather than HIV-1/AIDS. Fortunately, the accidental cures indeed brings hope for the future use of personalized gene therapy for AIDS.

### Hepatitis B Virus

**Figure 3:**
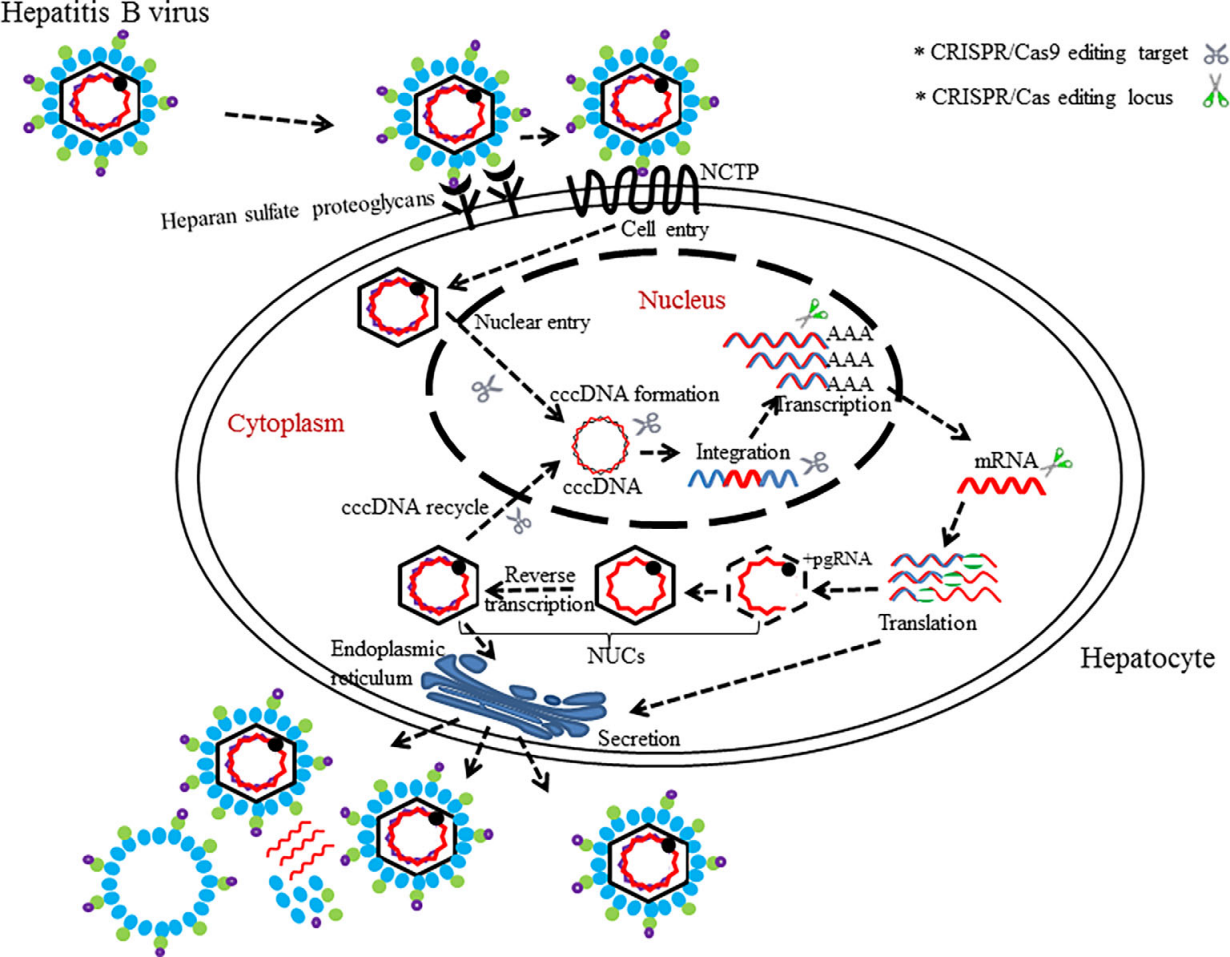
The life cycle of HBV with editing targets. HBV binds to surface receptors and enters the hepatocytes. Virus particle complete its process of growth and proliferation in host’s hepatocytes. CRISPR/Cas (or CRISPR/Cas9)-mediated disruption of the HBV life cycle can target several loci, which is necessary for the HBV life cycle.

The population figure of chronic HBV carriers in the world (350–400 million people (Seo and Yano, 2014) suggests that hepatitis B is still an important health problem (Trépo et al., 2014). Hepatitis B virus (HBV), of the family Hepadnaviridae (Locarnini et al., 2013), is a hepatotropic DNA virus that replicates by reverse transcription in host hepatocytes at the stage of RNA intermediates and can lead to relatively high frequent occurrences of liver cirrhosis and liver cancer in chronic HBV infectors (Lee, 1997; El-Serag, 2012). Taxonomically, eight genotypes (A–H) of the HBV genome have been identified, in which over 8% of the nucleotides differences are present between any two (Sunbul, 2014). Given that the chances of HBV-infected persons acquiring sustained viral response (SVR) or cure are small, novel and more effective regimens against HBV need to be develop (Nassal, 2015). The rapid growth of the CRISPR/Cas9 technology provides opportunities for new approaches in the prevention and treatment of HBV infectious diseases. As we know, the persistence of covalently closed cyclic DNA (cccDNA) of HBV is the major obstacle hindering the eradication of chronic hepatitis B (CHB) under current antiviral treatments such as nucleoside analogues (NAs) and interferon-alpha (IFN-α) (Emery and Feld, 2017). So far, gene therapies have become the promising potential treatment for HBV infections especially in targeting of cccDNA effectively and hold high promise for entering clinical applications after overcoming some technical barriers (Maepa et al., 2015; Bloom et al., 2018). Suppression of HBV infection in preclinical applications through gene editing platform ZFNs or TALENs have been reported by two research groups independently (Weber et al., 2014; Dreyer et al., 2016). In 2014, the ground-breaking work to use CRISPR/Cas9 system in countering HBV infection *in vitro* and *in vivo* was first investigated by Lin et al. (2014). Thereafter, several studies have utilized designed Cas9/sgRNA (or Cas9/multiplex gRNA) combinations to edit only one locus (which is usually in the conserved region of HBV genome) for inhibiting the viral replication and production successfully (Dong et al., 2015; Karimova et al., 2015; Liu et al., 2015; Seeger and Sohn, 2016; Zhu et al., 2016; Li et al., 2017; Scott et al., 2017; Schiwon et al., 2018; Kostyusheva. et al., 2019b). In order to enhance the silence effects for targeted genes, multiple research teams have worked on the applications of CRISPR/Cas9 for the simultaneous targeting and cleavage of several functional loci [e.g., surface antigen region, X gene, reverse transcriptase (RT) gene, and episomal cccDNA] in HBV genomes *via* cell cultures or mouse models (**Figure 3**) (Seeger and Sohn, 2014; Kennedy et al., 2015; Ramanan et al., 2015; Wang et al., 2015; Zhen et al., 2015; Sakuma et al., 2016). In addition to the CRISPR/Cas9 system itself, several other studies associated with the combination of CRISPR/Cas9 and other methods (e.g., different molecules or inhibitory systems) have also been developed for the purpose of eradicating HBV genomes (Wang et al., 2017; Zheng et al., 2017; Kostyusheva et al., 2019a).

Interestingly, a Cas9 variant called dead Cas9 (dCas9) has also been demonstrated to inhibit replication of HBV without dissection of HBV genome (Kurihara et al., 2017). It is noteworthy that one study was conducted recently to investigate a potent inhibitor of NHEJ named “NU7026,” which prevented the degradation of cccDNA mediated cleavages by CRISPR/Cas9 (Kostyushev et al., 2019). This study provides a verification methodology for the activity of CRISPR/Cas9 in destroying HBV genome.

Similarly to ZFNs and TALENs, there also exists a concern of viral escape mutants when there is therapeutic application of CRISPR/Cas9 systems in HBV-infected cells (Schinazi et al., 2018); despite of all these, nucleic acid editing tools could generate desired mutations on the target DNA (Pattanayak et al., 2013).

In summary, these artificial models (cell models or animal models) are only the simulations of persistent HBV infection in human hepatocytes and do not completely represent the actual HBV infection *in vivo*. These related studies do highlight the potentials of cccDNA disruption by endonuclease Cas9 protein in *in vitro* cells and *in vivo* mouse models. Nevertheless, additional studies are needed to ameliorate the CRISPR/Cas9 system so as to further destroy viral reproduction *in vivo* and to eradicate multiple HBV cccDNA copies residing in infected hepatocytes (**Figure 3**).

### Human Papilloma Virus

HPVs are small double-stranded DNA viruses belonging to the Papovaviridae family, with approximately 150 identified types already described (Nguyen et al., 2014; McBride, 2017). The HPV genome is roughly 8 kbp in length, encodes 9 or 10 open reading frames (ORFs) and includes eight early viral regulatory proteins (E1−E8) and two late capsid proteins (L1 and L2) (Ebrahimi et al., 2019). Since HPVs present epithelia tissue tropism (Harden and Munger, 2017), sexual transmission (Ryndock and Meyers, 2014), and oncogenic property (Moens, 2018), their important status between human diseases and public health must be emphasized. Continued high-risk type HPV (e.g., HPV-16 and HPV-18) infection is highly associated with the development of cervical cancers in women (Gupta and Mania-Pramanik, 2019). HPV can also initiate other kinds of anogenital cancer, head and neck cancers, and genital warts in men and women (Chen et al., 2018). Currently, there is no clinical cure for HPV infection that can achieve a satisfactory effect due to the ability of the virus to reduce their activity in a host cell to circumvent a host immune surveillance, which makes it extremely difficult to remove a viral genome from an infected host cell in a latency state (Lee, 2019). Based on the existing literatures, HPV-driven tumor formations have been mostly attributed to the HPV E6 and E7 oncoproteins, whose corresponding genes are regarded as two prime therapeutic targets in gene therapy (Moody and Laimins, 2010; Hoppe-Seyler et al., 2018). Theoretically, HPV E6 and E7 genes serve the function of suppressing cellular tumor suppressors p53 and retinoblastoma protein (pRB), respectively (Kennedy and Cullen, 2017). Therefore, overexpression of E6 or E7 induced by HPVs can cause malignant transformation of human cells with high probability through the activation of cellular oncogenes (e.g., *ras* or *fos*) (McLaughlin-Drubin and Munger, 2009).

**Figure 4:**
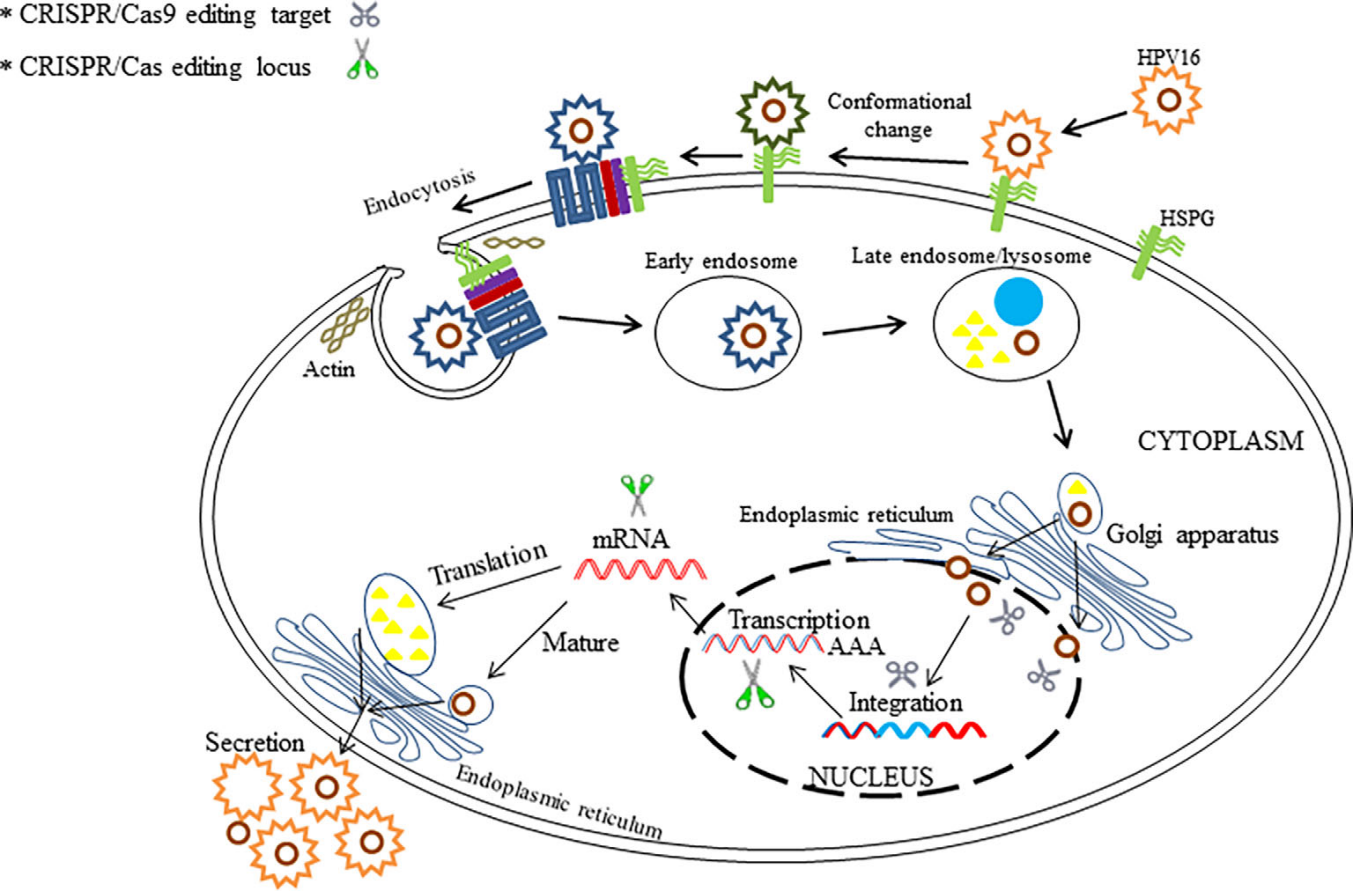
The illustration shows the potential editing and therapeutic targets in HPV life cycle by the use of CRISPR/Cas and CRISPR/Cas9.

There is still an urgent need to develop novel effective therapies for HPV-associated carcinogenesis, although many progresses have been made in different treatments for HPV. Now, the technology of CRISPR/Cas9-based gene therapy for HPV infection has come into being in recent years. So far, several articles have reported anti-HPV applications of CRISPR/Cas9 system for the purpose of disruption of the HPV genome (Hu et al., 2014; Kennedy et al., 2014; Yu et al., 2014; Zhen et al., 2014; Liu et al., 2016; Yu et al., 2017; Cheng et al., 2018; Hsu et al., 2018; Lao et al., 2018; Jubair et al., 2019; Yoshiba et al., 2019; Gao et al., 2020; Inturi and Jemth, 2021; Zhen et al., 2016; Zhen et al., 2020) (**Table 2**). Based on the investigations, the CRISPR/Cas9 approach has much development potential to act as an effective therapy for HPV-associated diseases in clinical settings. Herein, several editing targets of CRISPR/Cas (CRISPR/Cas9) in HPV life cycle are shown in **Figure 4**. Both for HBV and HPV, CRISPR technology is a novel method for the treatment of such viral diseases because it can fill in the technical gaps in drug therapy when a vaccine has already existed. However, CRISPR-associated technologies still need to be developed to improve the therapeutic effects.

**Table 2:** List of CRISPR/Cas9-based antiviral studies on targeting HPV.

Gene Editing Platform	Target Virus	Delivery Pattern	gRNA Target	Cell or Animal	Reference
CRISPR/Cas9 system	HPV-16	Transfection	E7	SiHa, Caski, C33A, and HEK293 cells	Hu et al., 2014
CRISPR/Cas9 system	HPV-18	LV transduction	E6, E7	HeLa cells, SiHa cells, 293 T cells	Kennedy et al., 2014
CRISPR/Cas9 system	HPV-16	Plasmids transfection	E6	SiHa and CaSki cells	Yu et al., 2014
CRISPR/Cas9 system	HPV-16	Plasmids and lipofectamine transfection	E6, E7	SiHa and C33-A cells, BALB/C nude mice	Zhen et al., 2014
CRISPR/Cas9 system	HPV-6, HPV-11	Plasmids transfection	E7	Human keratinocytes	Liu et al., 2016
CRISPR/Cas9 system	HPV-16	Plasmids and lipofectamine transfection	E6, E7	Siha and C33-A cells	Zhen et al., 2016
CRISPR/Cas9 system	HPV-18	Plasmids transfection	E6, E7	HeLa cells	Yu et al., 2017
CRISPR/SaCas9 system	HPV-16	AAV delivery	E6, E7	293 T cells	Hsu et al., 2018
CRISPR/hCas9 system	HPV Pseudotype Virus	Plasmids transfection	E6	SiHa cells, 293FT cells	Cheng et al., 2018
CRISPR/SpCas9 system	HPV-18	Micelle delivery; lipofectamine	E7	Hela cells	Lao et al., 2018
WT Cas9, FokI-dCas9	HPV-16, HPV-18	Liposomes	E6, E7	Mouse model, CasKi cells, HeLa cells, HEK293T, Jurkat cells	Jubair et al., 2019
CRISPR/Cas9 system	HPV-18	Plasmids; AAV delivery	E6	HeLa, HCS-2, and SKG-I cell lines	Yoshiba et al., 2019
CRISPR/Cas9 system	HPV-16	Plasmids transfection	E7	SiHa cells, Hela cells, nude mice	Gao et al., 2020
CRISPR/Cas9 system	HPV-18	Plasmids transfection	E6, E7	HeLa (CCL-2) cell lines	Inturi and Jemth, 2020
CRISPR/Cas9 system	HPV-16	Lipofectamine delivery	E6/E7	SiHa cell	Zhen et al., 2020

## Obstacles of CRISPR/Cas9 in the Treatment of Human Infectious Viruses

**Figure 5:**
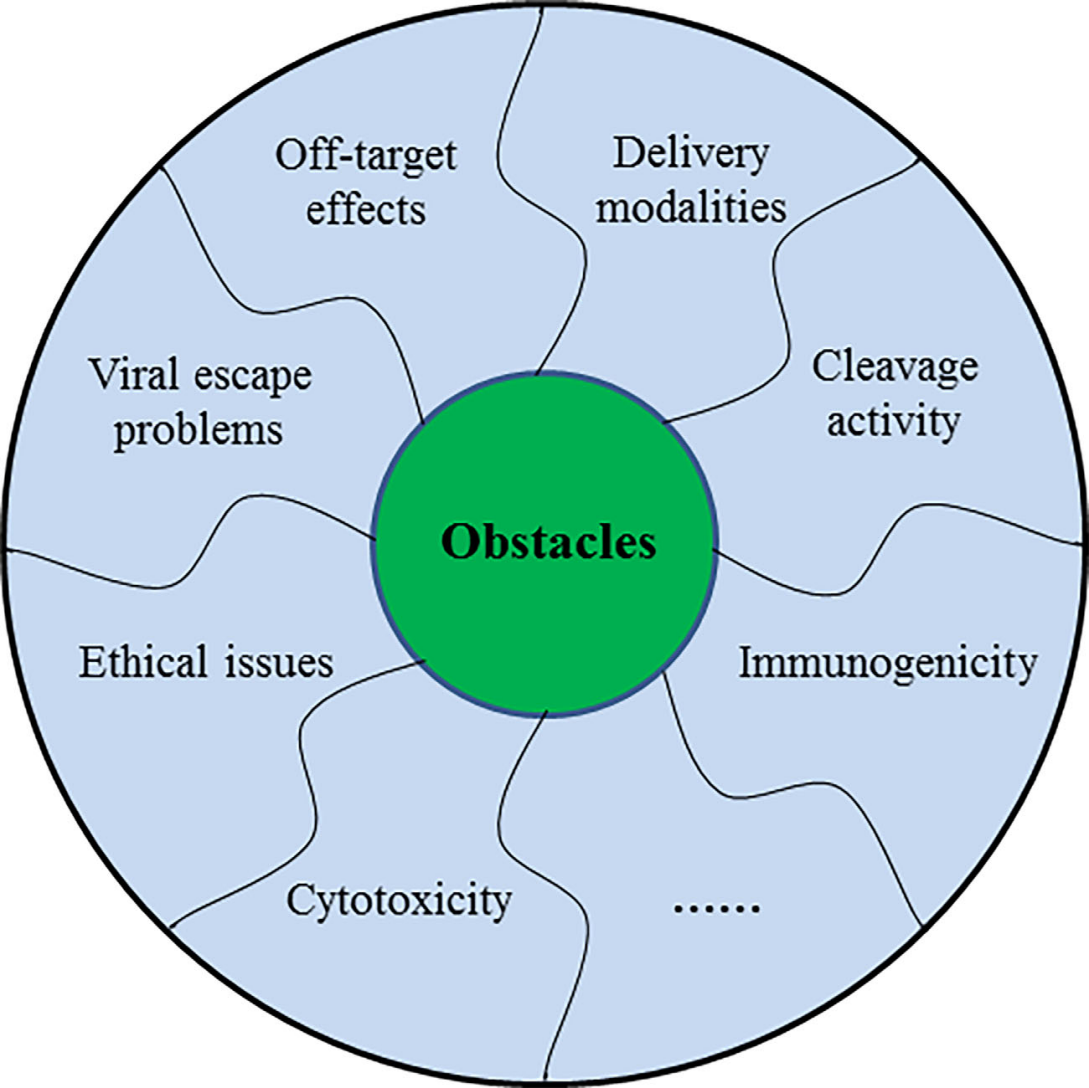
The schematic diagram showing several challenges of CRISPR/Cas9 in the treatment of human infectious viruses.

CRISPR/Cas9 system holds considerable potential for therapeutic applications of human infectious viruses *in vivo*, but certain questions have to be addressed before its use in clinical aspects. Generally, the off-target effect is the major concern associated with the use of this system. Other crucial challenges including delivery methods and strategies, Cas9 cleavage activity, resistance to Cas9/sgRNA system, viral escape problem, and ethical concerns still lie ahead (**Figure 5**).

### CRISPR/Cas9 Off-Target Effects

The CRISPR/Cas9 off-target concerns pose challenges for research advancements and therapeutic utilizations. Therefore, researchers have developed methods such as advanced versions or Cas9 nickases to minimize off-target activities (Bellizzi et al., 2019) and cytotoxicity (Wang et al., 2016). The two parts of CRISPR/Cas9 system, the optimizations of Cas9 proteins or the upgradations of gRNA, have equally contributed to the reductions in CRISPR/Cas9 off-target effects. On the one hand, many investigations have focused on reducing the unwanted off-target activities of CRISPR/Cas9 system (Eid and Mahfouz, 2016) through the amelioration of Cas9 nucleases. Recently, two versions of Cas9 variants termed “eSpCas9” (Slaymaker et al., 2016) and “SpCas9-HF1” (Kleinstiver et al., 2016) significantly optimize the CRISPR/Cas9 genome-editing toolbox with their own higher specificity and exceptional precision. On the other hand, it is very important to design special and compatible gRNA for the CRISPR/Cas9 systems. Nowadays, multiple website platforms are available for the optimal design of CRISPR gRNA (e.g., https://www.genscript.com/gRNA-design-tool.html and https://www.atum.bio/eCommerce/cas9/input). Besides, there are also other approaches whereby incorporation of chemical modifications into its structure improve gRNA stability and activity in the cell. Additionally, most CRISPR/Cas spacers that exist in bacterium naturally correspond to foreign nucleic acids (Hille et al., 2018), which precisely confer the bacterial immunity, but when this system works in animal cells, artificially designed CRISPR/Cas9 systems usually neglect this “implied condition” that can result in off-target effects, cytotoxicity, and cellular resistances. For example, Kim et al. reported that cytotoxicity caused by the tailored CRISPR gRNAs (5′-ppp gRNAs) triggers RNA-mediated innate immune responses in human and murine cells (Kim et al., 2018).

### Delivery Modalities of CRISPR/Cas9

Delivery mode perhaps remains the biggest bottleneck to gene therapy. To enhance gene editing efficiency, in addition to the improvements of CRISPR/Cas9 reagents itself, another key factor is the delivery methods of this gene editing system. More importantly, delivery methods that maximize efficacy and minimize immune responses still need to be developed (Schinazi et al., 2018). For CRISPR/Cas system, Cas9 nucleases can be delivered to gain access to the genome of the target cells in the form of DNA, mRNA, or protein, while the gRNA could be transferred in the form of DNA or RNA (Yin et al., 2017). Therefore, Cas9 protein and gRNA can be transported either together or separately, which provides much more options for choosing the vehicles. For example, a single adeno-associated virus (AAV) vector (AAV’s packaging capacity is ∼4.8 kb) usually cannot accommodate SpCas9 gene (4.1 kb) and its gRNA sequence. The solution to this problem is to use a small SaCas9 (3.2 kb) to take the place of SpCas9 or use two vehicles to separately transport SpCas9 gene and gRNA sequence.

The delivery patterns for CRISPR/Cas9 system are generally categorized as viral vectors or non-viral vectors, both of which have their own unique advantages and limitations (Nelson and Gersbach, 2016). Viral-based vehicles commonly include adenovirus, lentivirus, AAV, and retrovirus (Tong et al., 2019). Other viral carriers less in use for delivery still include herpes simplex virus and poxvirus (Jin et al., 2014). Non-viral delivery modes can be basically divided into two groups: physical methods [e.g., electroporation, microinjection, sonoporation, and hydrodynamic delivery (Mellott et al., 2013; Ibraheem et al., 2014; Fajrial et al., 2020)] and chemical approaches [e.g., lipid particles (Yin et al., 2014), polymer nanoparticles (Kirtane and Panyam, 2013), gold nanoparticles (Ding et al., 2014), and cell-penetrating peptides (CPPs) (Farkhani et al., 2014)]. Taken together, the proper choice of delivery tool is essential for the safety of gene therapy using CRISPR/Cas9 platform.

### Cas9 Cleavage Activity

To date, a growing series of wild-type and engineering Cas9 homologues and other CRISPR/Cas systems are expanding the gene-edited toolkits. However, Cas9 proteins with different species have distinct characteristics such as activity; scientists need to select the appropriate nucleases according to the research requirements and study protocols. For example, the SpCas9 enzyme is most commonly used for genome editing and genetic manipulation in eukaryotic cells while using CRISPR/Cas partly because of its high activity and comparative broad PAM compatibilities. Kim et al. listed 13 types of SpCas9 variants [wild-type SpCas9, eSpCas9 (1.1), SpCas9-HF1, HypaCas9, evoCas9, xCas9, Sniper-Cas9, and SpCas9-NG and the VQR, VRER, VRQR, VRQR-HF1, and QQR1 variants] for choice and made a comparison based on the activities, specificities, and PAM compatibilities (Choi et al., 2019). Eventually, the experimental results recorded on the overall activity could be ranked as SpCas9 ≥ Sniper-Cas9 > eSpCas9 (1.1) > SpCas9-HF1 > HypaCas9 ≈ xCas9 >> evoCas9 (Kim et al., 2020). Another example is that, when applying AAV as delivery vectors, small-volume genome-editing proteins with equal cleavage activity such as *S. aureus* Cas9 (SaCas9) or *Campylobacter jejuni* Cas9 (CjCas9), and other newly identified CRISPR/Cas enzymes may circumvent the limitation of packaging capacity (Doudna, 2020).

### Resistance to CRISPR/Cas9 (Cas9 Immunogenicity)

Curbing off-target activity has contributed immensely to the area of CRISPR/Cas gene therapeutics (Dolgin, 2020). Once the CRISPR/Cas9 system has been delivered into the target cell and being activated, there are limited means to lower or shut off its activity (Pawluk et al., 2016), which could raise new practical challenges and safety concerns to researchers. For example, excessive or prolonged Cas9 activity can exacerbate off-target effects. At present, some wild-typed Cas9-specific “anti-CRISPRs (Acr)” provide biotechnological tools that can be used to adjust the activities of CRISPR/Cas9 for gene engineering (Rauch et al., 2017). Recently, more than 50 anti-CRISPR protein families have been characterized, which provide various kinds of applications in genome engineering such as in posttranslational switches for control of Cas9 or dCas9 activity (Wiegand et al., 2020).

### The Problem of Viral Escape

The problem of viral escape has been a serious source of concern in the field of virus research. Many viruses possess the capability to escape or inhibit the effect of pharmaceuticals (e.g., interferon) in their evolution. Specifically in CRISPR/Cas9 applications, viruses can escape from these suppressions through the acquisitions of specific mutations at the target site that prevent gRNA binding without hindering viral replications (Binda et al., 2020). Using the HIV-1 as example, as far as we know, the RNA interference (RNAi) technology for the treatment of HIV-1 has already reached the clinical stage (Bobbin et al., 2015; Swamy et al., 2016). As observed with RNAi techniques previously, CRISPR/Cas9-based therapy of HIV-1 could also generate the self-replicated viral mutants (White et al., 2016). Recent studies reported that PAM sequence mutations have been shown to allow phage to escape CRISPR/Cas system (Bikard and Barrangou, 2017; Strich and Chertow, 2019). However, viral escape is not insurmountable if an appropriate gene editing treatment measures are taken.

### Ethical Issues

CRISPR/Cas9 technology is still in infancy stage, and many technical problems remain to be solved. However, the utilizations of CRISPR/Cas9 systems toward clinical applications will be confronted with some ethical questions. First, the misuse of this novel technology could likely create certain ethical controversies. Second, the safety induced by unwanted gene editing of CRISPR/Cas9 should be carefully improved and evaluated while clinically applying this system. However, in UK, scientists have gained license to edit human embryos with the use of CRISPR/Cas9 technology (Callaway, 2016), which potentially shows the technical strengths of this system in prospective clinical applications. Nevertheless, CRISPR/Cas9-associated clinics in the future must be strictly supervised with newly established regulations (Shinwari et al., 2017) so as to boost CRISPR gene editing technology to really serve humans.

Given the rapid progression of gene editing technologies, CRISPR/Cas9 is revolutionizing our ability to manipulate human genes and providing immense potentials and challenges for clinical trials. Hence, CRISPR/Cas9-based genomic methodologies will undoubtedly improve human life.

Howerver, CRISPR babies are currently not ready yet. As we know, significant progress has been made recently in CRISPR technology and has promoted the rapid development of biomedicine, agriculture, and animal husbandry. However, the off-target effect cannot be completely eradicated, the accuracy is not high enough, and the risks to growth are also unclear. At present, there is a worldwide consensus that *in vitro* gene editing research on embryonic development stage or germ line cells is allowed; CRISPR babies are expressly forbidden.

## Conclusion

Virus–host interaction is a fluctuant and persistent process within the infectious life cycle. The existence of several tricky viruses has led to the continuous upgrade of anti-virus approaches. CRISPR/Cas-based genetic targeting technology represents an alternative solution for treatment applications of virus-related diseases in the future. To date, CRISPR/Cas9 technology has already demonstrated many potential applications to human illnesses including genetic disorders, tumors, and infectious viruses. In addition to the aforementioned viruses, this technology is becoming increasingly powerful and have already been extensively applied into study on preventing and combating additional human viruses including Epstein–Barr virus (EBV), hepatitis C viruses (HCV), Kaposi sarcoma virus (KSHV), JC virus (JCV), and Herpes simplex virus (HSV). Moreover, CRISPR/Cas9 technology has been utilized not only in the treatment of viral infections but also in the investigations of cellular mechanisms of viral carcinogenesis.

In summary, continued efforts on developing CRISPR/Cas systems will expand the toolbox, which enables us to acquire a greater understanding of complex biological processes associated with hosts and viruses. However, the future use of CRISPR/Cas9 for gene therapies need substantial improvements and perfections before clinical applications.

## Author Contributions

HL, GL and XP wrote this manuscript. AD and LS help modify the manuscript. JH and TW direct the writing and support this project. All authors contributed to the article and approved the submitted version.

## Funding

This work was collectively supported by grants from the National Key Research and Development Plan (2016YFD0500600), Guangdong Provincial Science and Technology Plan project (2017B020207004), Fundamental Research Funds for the Central Universities (21618309), Innovative province construction project--special topic on fighting against novel coronavirus pneumonia epidemic (2020SK3044), Youth fund project of Hunan natural science foundation (2019JJ50681), Changsha biological resources sample bank establishment project (20200365) and Fund project of Hunan Provincial Health Commission (20200365).

## Conflict of Interest

The authors declare that the research was conducted in the absence of any commercial or financial relationships that could be construed as a potential conflict of interest.

## Publisher’s Note

All claims expressed in this article are solely those of the authors and do not necessarily represent those of their affiliated organizations, or those of the publisher, the editors and the reviewers. Any product that may be evaluated in this article, or claim that may be made by its manufacturer, is not guaranteed or endorsed by the publisher.
